# Disruptive Technologies for Achieving Supply Chain Resilience in COVID-19 Era: An Implementation Case Study of Satellite Imagery and Blockchain Technologies in Fish Supply Chain

**DOI:** 10.1007/s10796-021-10228-3

**Published:** 2021-12-03

**Authors:** Tuhin Sengupta, Gopalakrishnan Narayanamurthy, Roger Moser, Vijay Pereira, Devleena Bhattacharjee

**Affiliations:** 1 Indian Institute of Management Ranchi, Ranchi, India; 2 University of Liverpool Management School, Liverpool, UK; 3 Macquarie Business School, Macquarie University, Sydney, Australia; 4 Neoma Business School, Mont-Saint-Aignan, France; 5 Numer8, Mumbai, India

**Keywords:** Disruptive technology, Satellite imagery, Blockchain, Seafood supply chain, Resilience, Positive deviance, Pandemic

## Abstract

In supply chains where stakeholders belong to the economically disadvantaged segment and form an important part of the supply chain distribution, the complexities grow manifold. Fisheries in developing nations are one such sector where the complexity is not only due to the produce being perishable but also due to the livelihood dependence of others in the coastal regions that belong to the section of economically disadvantaged. This paper explains the contextual challenges of fish supply chain in a developing country and describes how integrating disruptive technologies can address those challenges. Through a positive deviance approach, we show how firms can help unorganized supply chains with economically disadvantaged suppliers by carefully redesigning the supply chain through the integration of satellite imagery and blockchain technology. With COVID-19 in the backdrop, we highlight how such technologies significantly improves the supply chain resilience and at the same time contributes to the income generating opportunities of poor fisherfolks in developing nations. Our study has important implications to both developing markets and food supply chain practitioners as this paper tackles issues such as perishability, demand-supply mismatch, unfair prices, and quality related data transparency in the entire value chain.

## Introduction

The outbreak of the COVID-19 pandemic has significantly stressed the importance of supply chain resilience. The pandemic has severely impacted the business environment across the globe and has raised the importance of crisis management mechanisms to deal with uncertainties of such scale. As the world witnesses’ lockdowns and restrictions, organizations have started taking steps for recovery and planning for the future. For instance, a recent survey revealed that with organizations in many sectors having suddenly been forced to shift to remote work, leaders face challenges in ensuring employee engagement, productivity, and connectivity (Sull et al., 2020).

Amid such transformations within organizations as a means to resume day to day operations, the importance of supply chain resilience and risk management has assumed greater prominence. According to a report on the World Economic Forum, businesses are faced with impacts of reduced supply, issues with disruptions in the logistics network, and hurdles in adhering to contractual agreements among others (Hedwall, 2020). Circumstances have necessitated supply chain decision makers to rethink how they should manage their supply chain and take strategic decisions to convert such uncertainties and ambiguities into competitive advantages (Craighead et al., 2020).

The complexities grow manifold in certain supply chains where stakeholders belong to the economically disadvantaged section and form an important part of the supply chain distribution. One such sector is fisheries in developing nations where the complexities are two-fold: a) the fish produce is perishable and hence the sale price is dependent on the perceived freshness from the buyers, b) livelihood is dependent on the business of fishery (applicable to those who are selling fish as a means to earn money) as well as consumption of the same in the coastal region (applicable to the large coastal population who are dependent on fish as their staple food) where majority of the populated are economically disadvantaged. In addition, the severity of risks goes up since the entire supply chain is unorganized in such regions. As a result, many fishery cooperatives have come up in the last decade to aggregate and strengthen the supply chain.

However, with COVID-19, the entire supply chain is burdened with uncertainty in the demand. This is due to many reasons. First, due to lockdowns people are not encouraged to step out and are asked to avoid crowded places such as fish markets. The last mile fish sellers do not have the infrastructure to reach all potential customers through door-to-door delivery service. Even if they are able to supply a portion of the same, the demand is only a fraction of what it used to be before COVID-19. Also, the different rules of lockdowns in different places are giving inaccurate demand signals to the upstream players. Further, people are apprehensive about buying food such as fish regularly because of the sanitation activities to be carried out on carry bags and clothes after they return to their homes. Due to this uncertainty and fear among the buyers, the supply of fish has been unpredictable (as demand is unpredictable) leading to shortage of food to a large population who are dependent on fish. This has a severe impact on the livelihood of fishermen and the population residing in the coastal regions in developing and emerging nations. Based on the research conducted by Numer8, more than 800 million depend on fish globally. Around 3/7th of the people in this planet depend on seafood as their main source of protein. Approximately, 44% of the global population depend directly or indirectly on oceans for their livelihoods.

Given this complex nature of the fish supply chain and significant uncertainty introduced post the outbreak of COVID-19, the objective of this paper is to study the role and present the impact of disruptive technologies in tackling risks in fish supply chains in developing countries. Disruptive technologies refer to those technical interventions that ‘tend to be used and valued only in new markets or new applications’ and ‘they generally make possible the emergence of new markets’ (Bower & Christensen, 1995; Liu et al., 2020; Mahroof et al., 2020). Disruptive technologies can replace the existing mainstream technology in unexpected ways as they have often been simpler and usually easier to use and handle (Dhillon et al., 2001), making them economically and operationally appealing to managers. Some of the examples of disruptive technologies include big data (Nagendra et al., 2020a, 2020b), artificial intelligence (Rodríguez-Espíndola et al., 2020), blockchain (Wamba et al., 2020), 3D printing (Mohr & Khan, 2015), Internet of Things (IoTs) and smart robots for automation (Goldsby & Zinn, 2016).

The literature on supply chain resilience during COVID-19 is very scant and emerging due to the close proximity of the pandemic. Although supply chain resilience has been well understood from the perspective of definition and its relevance to different context, very little is known on how disruptive technology could be integrated in the supply chain to improve supply chain resilience is missing in the current literature (Casado-Vara et al., 2018; Ehrenberg & King, 2020; Zhu et al., 2021). This is important as the technology proves to be very effective in supply chains that are not matured in terms of traceability. Also, the need to understand the same is more justified when economically disadvantaged stakeholders are involved and the commodity is food which directly impacts the livelihood of developing nations. As a result, the importance of trust and traceability in the food supply chain (Choe et al., 2009; Lai et al., 2013) is key for supply chain resilience.

The traditional fish supply chain consists of fishermen who venture out to deep seas and backwaters to locate fish, and then return to the shore where they would sell their stock to intermediaries who would then transport it to different parts of the region and sell it to local distributors and retailers. Major problems with the traditional model are as follows: a) high search cost while in the sear for catching fish, b) little knowhow of demand from consumers, and c) absence of trust from consumers with regard to fish quality due to lack of transparency in upstream activities (e.g., catchment source, storage, packaging). As a result, the adapted supply chain integrating disruptive technologies will not only help fishermen in finding the right kind of fishes in deep seas but also ensure that consumers are informed about the traceability of produce.

In this paper, relying on a single in-depth case study approach and interacting with Numer8 as our industry case organization, we investigate, through the lens of positive deviance, how Numer8 integrated two disruptive technologies, namely satellite imagery and blockchain, into fish supply chain. The positive deviance approach helped us understand how innovative entrepreneurship such as that of Numer8 integrated different stakeholders in a digital platform and through the help of different disruptive technology implemented in the value chain modified the traditional model of fish supply chain in India. One of the major contributions of this paper is to observe how firms adopt positive deviance approach while innovating in developing nations and addressing the challenges and bottlenecks associated with it. By doing so, we specifically try to answer the following research questions:

- RQ 1: How can disruptive technologies be integrated into fishing supply chain for alleviating the identified bottlenecks during the global pandemic?
- RQ 2: How these disruptive technologies impact supply chain resilience?

We intend to highlight the role blockchain and satellite imagery can play in improving the traceability and resilience of fish supply chain under the influence of the global pandemic. Traceability followed by trust through blockchain technology would ensure setting of right prices at the consumer level and also reduce the number of intermediaries who take advantage of the lack of knowledge of customers regarding perishability of produce. This would shift the benefit to the economically disadvantaged fishermen and also would help these fishermen to accurately predict demand due to reduction of information asymmetry in the supply chain. Resilience as an overall outcome of the adaptive supply chain ensures better preparedness in emergency situations such as the global pandemic. Our findings highlight social and economic benefits to fishermen, improved consumer trust towards sellers, better management of fish supply chain due to transparency and traceability and most importantly improving the resilience of the supply chain in such emergency situations.

The remainder of the proposal is as follows: In the next section, we summarize the relevant literature, while triangulating the research gap in the literature. In the third section, we explain the theoretical framework on which we base our research context. Section 4 outlines our methodology that we adopted for this study. Section 5 documents the findings of our study. Section 6 presents the discussion section which links the findings of the study with the theoretical framework adopted. The last section concludes our manuscript and explains key research, practice and social implications of our study and also point out the limitations and future directions for this manuscript.

## Literature Review

We converge on our research gap by reviewing literature from supply chain resilience and COVID-19 (Ivanov & Das, 2020; Ivanov & Dolgui, 2020; Schatteman et al., 2020), and disruptive technologies contributing to supply chain resilience (Chen et al., 2019). These literature streams are chosen in order to triangulate our research gap and argue our motivation for conducting this research as stated in the previous section. Since our broad objective is to address how disruptive technology leads to supply chain resilience during emergency situations such as COVID-19, we therefore pivoted supply chain resilience literature and tried to understand the findings in the past literature both in terms of its linkage to technology such as Blockchain, satellite imagery and also its relevance in response to COVID-19.

### Supply Chain Resilience and COVID-19

The first stream of literature on supply chain resilience and COVID-19 investigated the role of risk management in supply chains in mitigating the disruptions caused by the global pandemic (El Baz & Ruel, 2021). It emphasized the role of dynamic resources and risk management practices as key enablers towards effective mitigation strategies. This was probably an extension to the discussion on how supply chain can survive in a changing environment and whether the concept of supply chain agility be integrated for building supply chain resilience (Ivanov, 2020). There may be instances where supply chain resilience capabilities may be inefficient. The current pandemic has revealed that a timely deployment of such assets and capabilities is difficult which in turn questions the value proposition of resilience assets. The AURA (Active Usage of Resilience Assets) framework tried to understand how resilience assets could be better utilized as an active and continuous component of decision making rather than a passive activity deployed only to protect against severe events such as the current pandemic (Ivanov, 2021).

We notice sector specific studies on the resilience dimension during COVID-19. For instance, there is one study which documented the lessons from automobile and airline sector and highlighted supply chain resilience dimension in wake of COVID-19 (Belhadi et al., 2021). SME’s was one of the severely affected sectors especially in developing countries. The literature recommends reactive strategies for SME’s in responding to COVID-19 by providing guidance based on the characteristic of the organization within that sector (Ali et al., 2021). The characteristics are based on time (response time needed to react) and the cost associated with the response as a reactive strategy to the current global pandemic. We also witnessed that food sector has been significantly impacted due to COVID-19 especially in developing markets where the supply chain visibility is low and as a result, demand and supply shocks have been prevalent (Nordhagen et al., 2021). There is a need for innovative and targeted approach for food supply chains that are shorter and smaller operational complexity in comparison to national food supply systems (Thilmany et al., 2021). Hence, local responses in the food supply chain systems may vary with the national policy responses to the pandemic. While economies of scale and scope offer advantages to the players in the food supply chain in normal scenarios; there is a need to make supply chains more flexible and adaptable in order to be more resilient (Hobbs, 2021). Therefore, there is a need for increased automation and digitalisation in the food supply chain for better resilience. Unfortunately, due to the recency of the global pandemic, none of the studies to the best of our knowledge document how specific firms in specific sectors should utilize technology in response to resilience. Our study specifically tries to address this gap by highlighting how firms could integrate technology in the food supply chain to increase supply chain resilience.

### Supply Chain Resilience and Disruptive Technology

In the second stream of literature on disruptive technologies resulting in supply chain resilience, the need of IT use with both customers and suppliers in building resilience in supply chains has been well emphasized in the literature (Gu et al., 2020). One of the critical discussions on resilience is emerging on the concept of Blockchain technology (Min, 2019). One of the advantages of blockchain technology is that it builds trust, collaboration and resilience in a supply chain setting (Dubey et al., 2020). However, one of the studies pointed out that firms should be careful while implementing blockchain in a supply chain (Vivaldini & de Sousa, 2021). The study lists out the factors, in a blockchain driven intervention, that could negatively impact the connectivity between supply chain actors (during the intervention) and thereby supply chain resilience.

Another outcome of deploying disruptive technology (IoT, Satellite Imagery, digitization) in today’s context is the generation of big data through these technologies. The question always arises whether such data could be used to improve resilience. There is very little evidence in the literature that establishes this relationship across different supply chains. With Chinese manufacturing firms, there is empirical evidence that big data has a positive impact on supply chain resilience by improving overall supply chain visibility (Zhang & Zhao, 2019). However, this relationship is negatively moderated by supply chain complexity. Further, it has been argued in one of the technical notes that big data can be harnessed to transform the overall capabilities in supply chains in order to meet external shocks such as the terrorist attack, financial crisis etc. (Meriton & Graham, 2017). Extending on the same arguement, enterprises should deploy IoT to achieve resilience in the supply chain (Cui, 2015) in light of effectively responding to environmental changes such as loss of critical supplier, an act of terrorism or a fire accident in the production facility (Al-Talib et al., 2020). Different big data analytics driven infrastructural capabilities have different impact on the supply chain resilience development. Hence, it is important to understand that appropriate technological development should be carried out at different points in the supply chain (Mandal, 2020).

While carefully observing the literature on disruptive technology and resilience, we found that very few studies discuss how disruptive technology specifically addresses the resilience dimension in the supply chain. On one side, it may be reasonable to argue that blockchain is important for supply chain resilience, however, on the other side, none of the studies looked specifically on how blockchain and/or other disruptive technology can improve the resilience from a firm level perspective. This is important as supply chain characteristics differ across sectors, boundaries and more importantly on the way stakeholders are involved in the overall supply chain structure. None of the studies focused on a specific supply chain, the role and impact of disruptive technology in emergency situations such as COVID-19. We therefore attempt to fill this gap by highlighting the power of disruptive technology, in our case it is the blockchain with the help of satellite imagery, in improving supply chain resilience and also its relevance to livelihood improvement efforts in food supply chains in emerging nations.

## Theoretical Framework

We are using the concept of positive deviance to understand our research context (Albanna & Heeks, 2019; Goode, 1991; Sagarin, 1985). We often observe that certain individuals, communities or actors in the value chain use non-traditional practices and/or exhibit uncommon behaviours in order to achieve better solution to the problems that they are facing (Pascale et al., 2010). These solutions are often effective in comparison to their peers and the individuals or entities that adopt these solutions on a large scale are known as the “positive deviants” (PD) and the process of adopting these solutions are often termed as the PD approach. Despite the effectiveness as an important problem-solving approach, PD approach has not found great prominence other than public health (Baxter et al., 2016; Lapping et al., 2002; Lawton et al., 2014). Even in the genre of sports, athletes’ over-conformity to sports ethic can be seen through the lens of PD (Hughes & Coakley, 1991). Among the recent papers, we find PD is used as a lens to understand employee engagement (Sharma, 2021).

We have very less evidence on the application of PD in food sector or related areas. For instance, we observe improved farming systems, through the PD approach, by redesigning the existing infrastructure in resource-poor contexts in Bihar, India (Toorop et al., 2020). Also, there are couple of studies on food safety education programs through the PD approach (Feng et al., 2016; Whited et al., 2019). Neffa-Creech et al. (2020) studied, through the lens of PD, the adoption and use of food and nutrition app in low-income Latino homes.

By investigating the reliance on PD approach in the past research, we make two observations. First, PD approach has been well documented in developing market context (Albanna & Heeks, 2019). Second, there is reasonable evidence to point that PD approach is gaining popularity in agricultural or food sector with specific emphasis to food safety (Feng et al., 2019). In addition, PD approach is gaining prevalence in social sciences and cross disciplinary research. Critiques have often recommended that PD approach works in situations that involves specific technical challenges (Albanna & Heeks, 2019). It does not work in situations where there is a need for learning and behavioural driven change from different stakeholders (Nel, 2018). Also, literature has argued that innovators have used the PD approach in solving tough problems across the globe (Pascale et al., 2010).

We, therefore, intend to approach the problem from a PD perspective. The fundamental difference from past literature on PD approach and our paper is that we approach the issues from a firm level perspective whereas most of the literature on PD, base their arguments on individual actions or actions taken by communities to address a given problem. In our case, we document how a firm addresses the challenges of the fish supply chain in a developing country by utilizing disruptive technology that was already present in the market in different supply chain. PD approach is most suitable as the entity uses a different approach with the help of resources (disruptive technology and stakeholders) that were already present in the value chain. The approach points out how disruptive technology, if properly channelized in the right way, can address critical issues in the supply chain including resilience, social responsibility which is of paramount importance in the present context where different stakeholders are impacted from the current global pandemic. This approach not only explain how firms adapt to challenges in emerging economies but also highlight how the resource, which in our case is the technology and the different stakeholders involved in the business, is utilized, integrated in a manner that contributes to the objectives of the firm, which in our case is the supply chain resilience. We therefore, believe that PD approach successfully demonstrates the research objectives our paper and contributes to the theoretical understanding on how firms could use this approach for addressing the challenges in developing nations.

## Data and Methods

We explain our research context in depth followed by the case study methodology being adopted and the rationale for it. While we explain the research context in detail, we wish to position it from three perspectives: 1) seafood ecosystem across the globe, 2) fish supply chain issues in developing nations and the impact of COVID-19, 3) technology intervention and its potential implications to different stakeholders.

### Research Context

The global seafood supply chain allows seafood from across the globe to reach our plates. Even though that sounds exciting, a recent study by Oceana, a marine conservation non-profit found 20% of seafood are mis-labelled (Oceania, 2019). Apart from mis-labelling, the biggest challenge for the food industry is to ensure food safety, lower food fraud and at the same time keeping low operating costs. Food safety and quality is vital for not only the seafood consumer but also for every handshake in the value chain. The World Health Organization estimates that almost 1 in 10 people become ill every year from eating contaminated food (WHO, 2020). The Center for Disease Control estimates that 48 million get sick from contaminated food every year in America alone (CDC, 2020). The transparency needed to adhere to the cyclical economy ensuring a fair-trade model is missing from the present-day seafood industry (McClenachan et al., 2016).

A typical food supply chain consists of five basic steps: 1) Supply of raw materials 2) Production 3) Food Processing and Packaging 4) Storage 5) Distribution (wholesale and retail). With specific reference to the fishery sector, the only characteristic that is common to any geographical region is that fish is a perishable produce and hence storage (cold storage) is an important part in the supply chain. Similarly, one differentiating factor in the fish supply chain in comparison to the food supply chain is that catching fish produce in deep seas and backwaters is a challenging task and once caught, there is no formal production process involved which is not the case for the food or agricultural sector where the certain items are processed for further consumption in the downstream value chain. Once the fish produce reaches the shore, it is then sold to different wholesalers which again get distributed to different retail market.

In developing nations, most of the fisherfolks belong to the economically disadvantaged section (FAO, 2005) of the population and due to many middlemen, the final retail price to the end consumer is often expensive and the share of the revenue does not get transferred to the fisherfolks. Also, the quality of fish is questionable as information asymmetry takes dominance due to multiple middlemen and absence of cold storage units across the entire value chain. Further, a large section of population with close proximity to vast coastline in developing nations such as India, Indonesia, Thailand, Myanmar, etc. are often dependent on fish supply for livelihood. Hence, any supply disruption (such as due to COVID-19) not only impacts the poor fisherfolks by lowering their earning opportunities but also severely impacts the livelihood for many since fish is a staple food for many consumers in the coastal region and due to such disruptions, supply is severely impacted as there is uncertainty in the demand. The global pandemic also taught us that demand of groceries including fish produce can fluctuate significantly putting a lot of economic stress on fisherfolks and middlemen due to the perishability nature of the product. Hence, resilience is important and can be achieved through greater transparency and visibility in the supply chain.

However, visibility and transparency as a capability would only succeed when all stakeholders in the supply chain stand to benefit (irrespective of the global pandemic being present). The benefit should be tangible to all and owing to the supply chain being unorganized, the upstream supply chain players should be able to do something differently (a positive deviance approach) in order to be more resilient and at the same time competitive in the market. Enterprises have adopted disruptive technology for different purposes. Often it is difficult to adopt, implement and accrue benefits of a digital transformational journey in unorganized markets such as the fishery sector in developing nations. We try to understand how such complexities (supply chain and disruptive technology) can be tackled in developing nations with specific reference to the fish supply chain especially in the wake of the current global pandemic.

### Data Collection and Analysis

We adopt a case study approach for our paper because our focus is to understand the “how” and “why” questions from our research paper especially when one cannot control the behaviour of actors in the study (Yin, 1998). The rationale for adopting a case study approach is to understand and explain an environment which is less researched and, in some sense, unique to the impact of COVID-19 in developing and emerging nations (Siggelkow, 2007). We conduct two-phase exploratory research (Baxter & Jack, 2008). In the first phase, we conduct an in-depth case study of an Indian firm that has implemented blockchain and satellite imagery to fishery cooperatives in countries such as India, Sri Lanka, Philippines among others. The first phase would help us to understand the bottlenecks in fishing supply chain in developing nations. Also, we would document how the fishing supply chain responded to global pandemic, given the identified bottlenecks. In the second phase, we analyse how disruptive technology is designed and integrated to respond to identified bottlenecks. In this regard, we conducted discussions with executives and employees involved in the case organization. Also, we investigate how such technologies have impacted the resilience of the involved supply chains in the process. The social dimension of such technological innovation is also captured in the results section.

### About the Organization

Numer8 was incubated in India by Unltd India and in Prague by European Space Agency. The organization’s strength lies in building end to end intelligent applications with the potential of affecting millions across the globe. They are a team of data scientists, data architects and cloud engineers and GIS analysts, marine biologists and professional fishers with a cumulative experience of more than 120 years. The CEO of the organization has more than 15 years of work experience as a data scientist and the CTO has more than 15 years of experience in big data architecture. The digital platform offered by this organization has a subscription from more than 1700 fisherfolks and more than 3500 seafood buyers. The case organization got featured by a leading newspaper in India for helping more than 600 fisherfolks increase their net income during COVID. It was awarded among the top three global innovations in UN’s World Food Programme in February, 2019. It is one of the top five global companies selected by Luxembourg Space Agency. The digital platform aims for sustainable, traceable and profitable seafood supply chain. It has also been recognized by leading websites and news channels such as Forbes India, and Mongabay, among others. Numer8 is suitable for achieving the objectives of this research for various reasons. First, it is one of the first few companies, to the best of our knowledge, which have successfully implemented disruptive technology in the fishery sector given the fact that this sector is severely impacted by COVID-19 in any developing country. Second, the context of India amplify the relevance in terms of it being severely impacted by COVID-19 (more than 311,421 fatalities and 27,157,795 cases as on May 26, 2021) and therefore its impact on the large population from the perspective of livelihood. Third, the findings of the same can be taken forward to other similar emerging market context given the fact that Numer8 is already working with other developing nations for similar interventions in the fishery sector.

## Results and Findings

We divide the section into three parts. The first part document the observation related to the food supply chain and the contextual bottlenecks in the presence of global pandemic. The second part highlights Numer8’s disruptive technologies and how the organization managed the entire supply chain through their e-commerce business model. Here, we highlight how the organization dealt with the bottlenecks mentioned through the PD approach. The third section explains how supply chain resilience would be achieved through the integration of these technologies in the presence of similar shocks in the future.

### Bottlenecks and its Impact during COVID-19

**Fig. 1:**
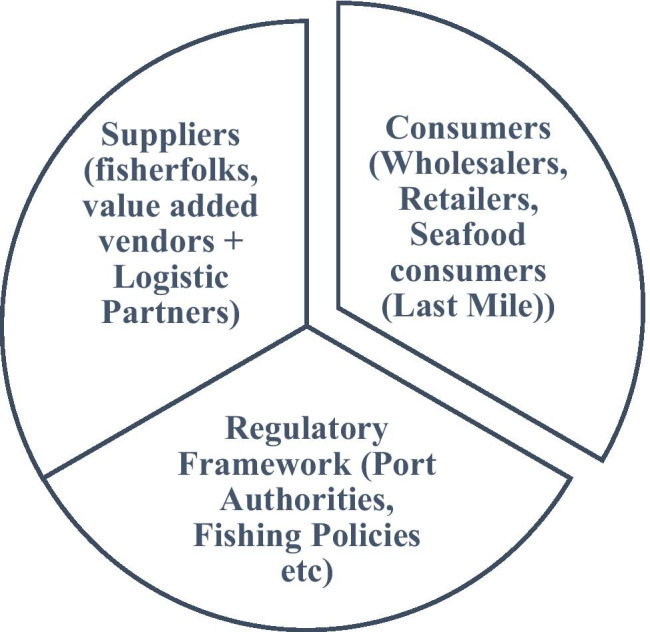
Seafood ecosystem

The sea food supply chain is highly disintegrated. This sector has been neglected for years due to the supply chain being primarily unorganized. Fisherfolks have been suffering financially due to 1) high search cost 2) high storage cost due to perishability 3) unfair prices due to middlemen. Similarly, buyers are suffering on the quality of fish especially the end consumers since many hands are getting exchanged by the time it reaches the final stakeholder in the supply chain. The ecosystem of fishery sector involves many stakeholders and compliances such as fishing policies, port authorities, fish sellers and buyers, fisherfolks, seafood consumers (last mile stakeholder) and value-added vendors such as those who produce ice boxes for storage and distribution etc. Figure 1 presents the schematic diagram of the seafood ecosystem.

In the presence of COVID-19, developing nations such as India was subjected to both state-wise and nationwide lockdowns which included strict rules for mobility, assembly and also localized time range under which the shops were allowed to transact with the consumer. In areas where consumers are dependent on fish, consumers started to buy fish in large quantities in anticipation for supply shocks in the subsequent days. On the other hand, big retailers who used to sell cold storage fish in supermarkets reduced their inventory in anticipation of lower demand since these supermarkets were allowed to open only for a certain time duration. With localized small fish retailers (fish sellers in fish markets), they had limitations for cold storage and therefore were scared whether their inventory will get sold as people were reluctant to come out and buy fish from the fish market. Hence, there was incomplete information in last mile transaction between buyers and sellers. As a result, the impact of bullwhip effect was high leading to confusions about demand and supply information. The impact in terms of price was severe as certain fish were sold at a very high price in the retail market due to fear of unavailability. At the same time, last mile sellers incurred huge losses due to perishability, and low sale price due to last moment sale of fish perishing in the inventory. Consumers were sceptical about the quality of fish and fisherfolks were sceptical about demand generation and final sale value of their produce. This problem was prevalent even in pre-COVID times; however, the global pandemic underlined the lack of resilience in the supply chain and the long-standing issues of quality, traceability, transparency and fair prices. Numer8’s digital platform, supported by the disruptive technologies, addressed these concerns through their e-commerce business model.

### Disruptive Technology and Business Landscape

We document the entire business and technological landscape of Numer8. While doing so, we highlight the role of disruptive technologies and its integration in the value chain. Also, we would understand how Numer8 as an organization adopted a PD approach to address the challenges of the fishery sector during COVID-19.

In relation to the bottlenecks mentioned above, Numer8 introduced the digital app platform, which they termed as the “OFish”. OFish is a suite of services that helps fisherfolks spend less money in the value chain and as a result end up earning more in the process. The services included supporting fisherfolks to efficiently identify fishing zones in the sea, trading the sourced fish with potential buyers, delivering the traded fish to the buyers which includes tracking and tracing the produce with quality monitoring at real time, and offering an opportunity to support their livelihood with access to microfinance through different intermediaries. Hence, the primary stakeholders are fisherfolks and buyers.

**Fig. 2:**
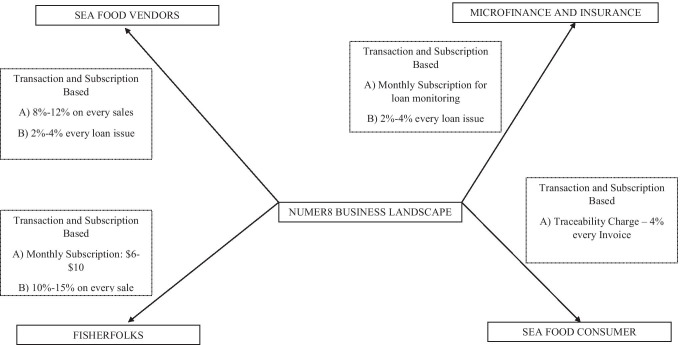
Business landscape of Numer8

The secondary stakeholders are seafood vendors which include logistic partners, supply of cold storage boxes, supply of nets; and microfinance institutions that provide loans to fisherfolks and other stakeholders in the process. Needless to say, the government also stands to be a tertiary stakeholder as it involves regulatory clearances for deep sea fishing and other compliance related formalities. However, we would not go into the details of how developing nations interact with government agencies in relation to compliance issues as it is outside the objectives of this paper. We present the entire business landscape in Fig. 2. Numer8 is the central player that ensures that all stakeholders come together and join the platform. Hence, it operates as a transaction-based model where it receives commission for every transaction made by different stakeholder in terms usage of the platform (example: fisherfolks, seafood vendors) and its services and/or in terms of the sale value of the transaction with different stakeholders (Example: microfinance institution).

**Fig. 3:**
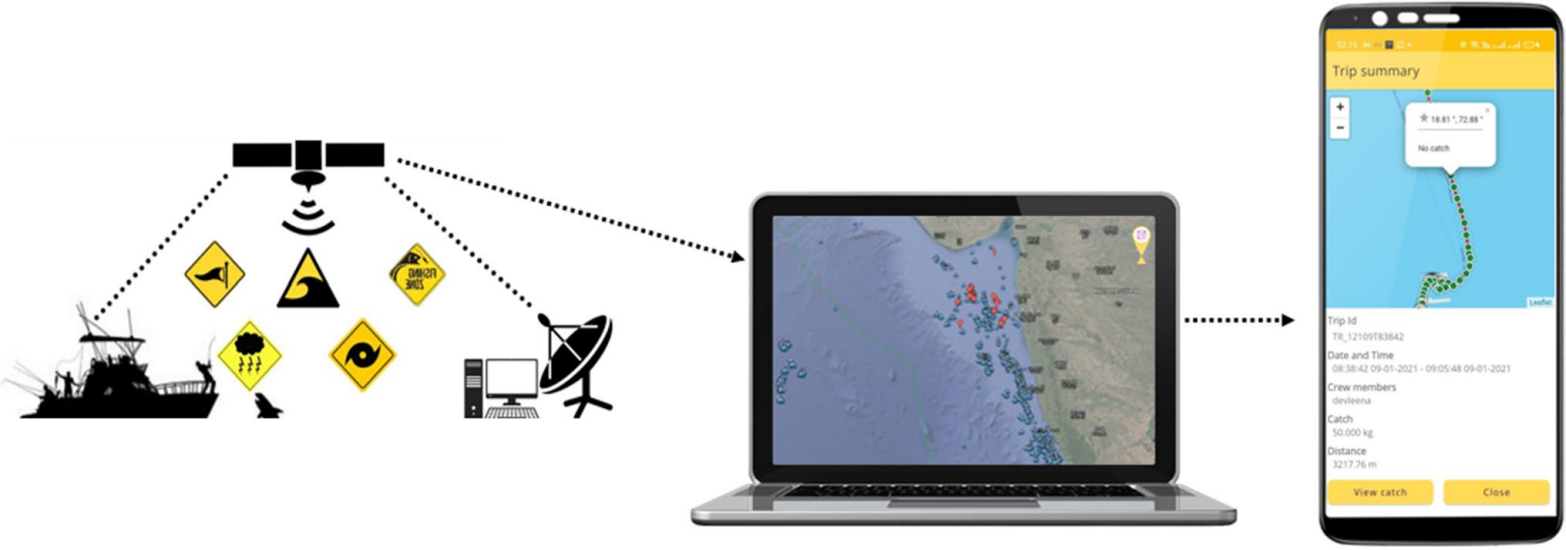
Tracking and tracing of fishing boats

The first use of disruptive technology is the satellite imagery (Disruptive Technology 1) and the flow of information to fisherfolks through the digital platform. Fisherfolks are provided with navigation (through app) with hyper local real time weather data, real time water quality data and trip and fish logs. The information includes zones of fishing where they should navigate so as to reduce time for search, hence reducing search cost. In addition, to reduce their non-value-added activities during navigation, information such as wind speed, wind direction, and tidal current (includes cyclone warning) are provided so that inefficiencies due to navigation is reduced leading to less wastage on fuel and time. This is expected to reduce their operational cost by almost 35%. With the help of GPS and other IoT devices mounted on the boats, the platform is able to track the movement of fishing boats in the region (as captured in Fig. 3). This data is often given to port authorities in case these boats come in close proximity to “no-go” zones as declared by the government. Also, such monitoring activities reduces chances of illegal fishing, helps rescue teams in case of any such emergency.

**Fig. 4:**
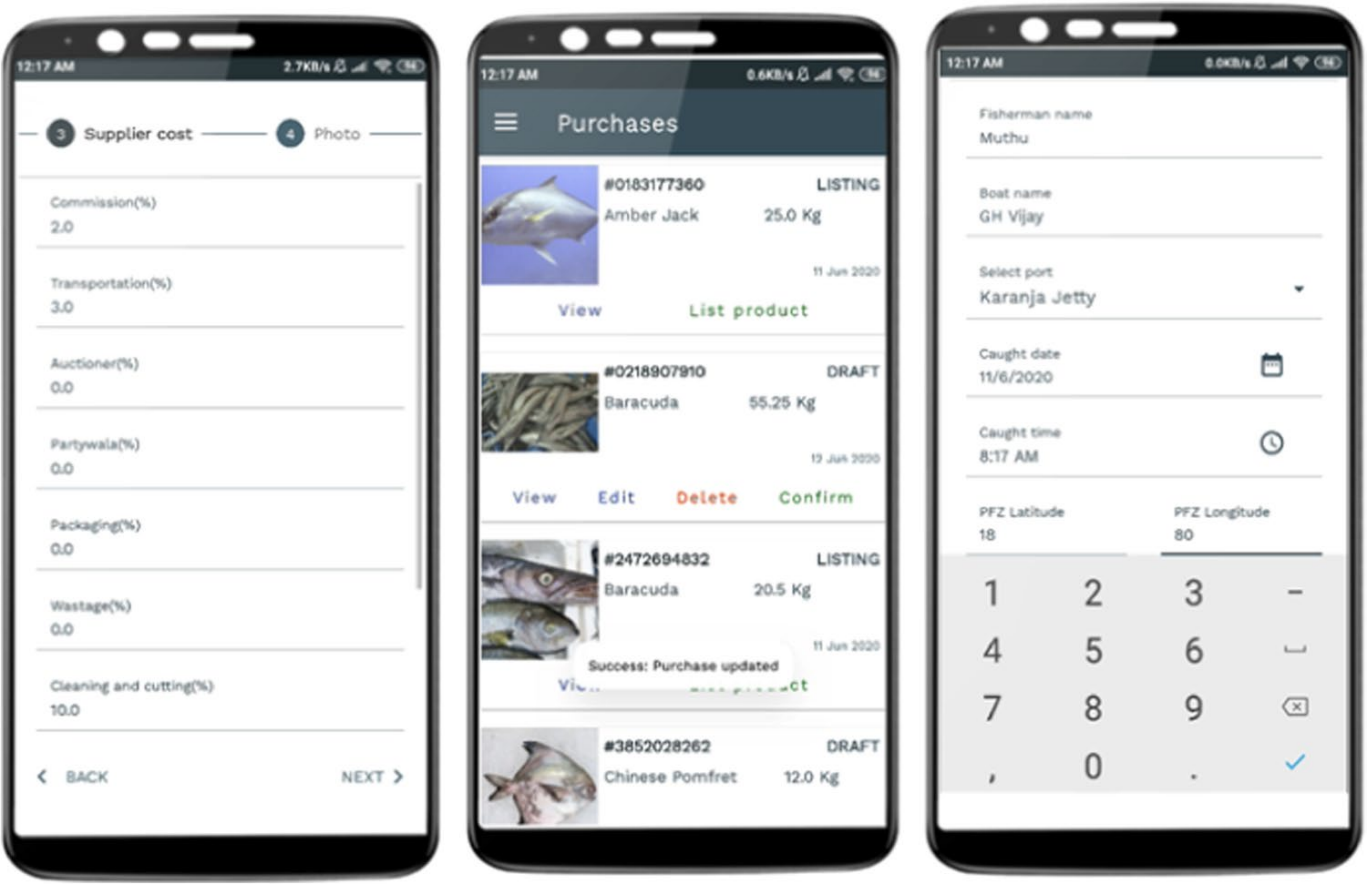
Digital App – During Sales

**Fig. 5:**
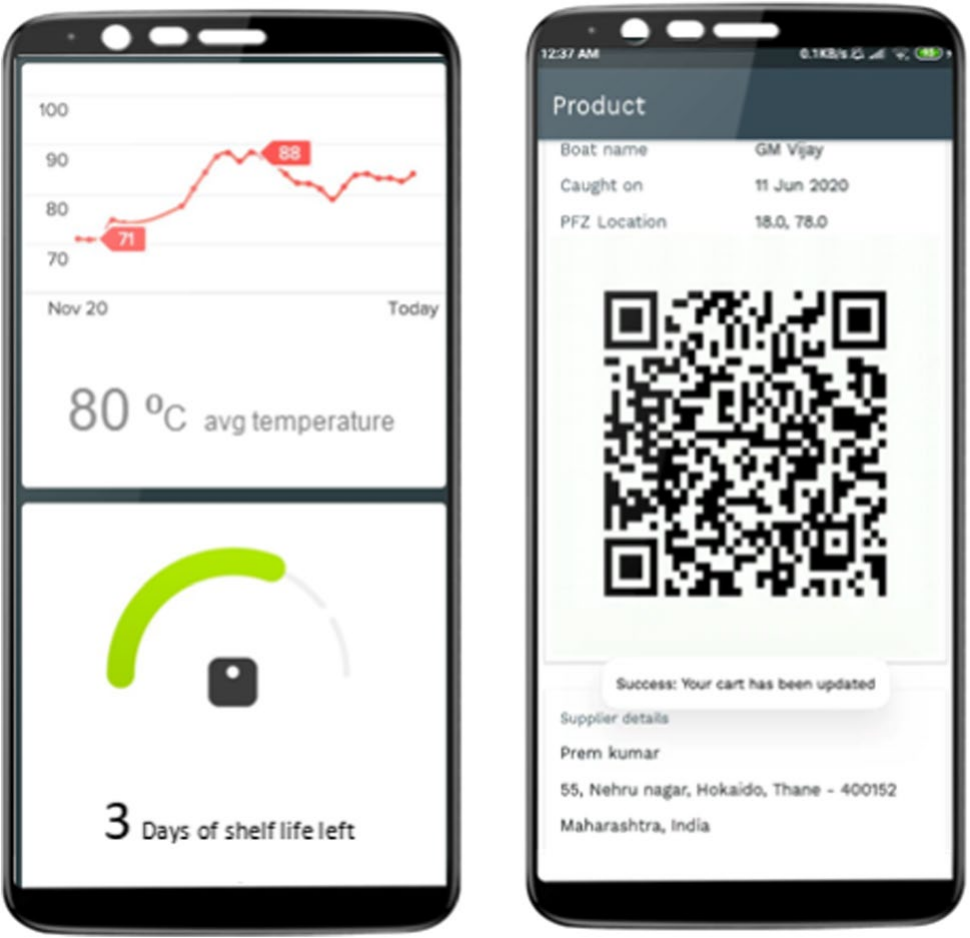
Tracking and food safety checks during delivery

Once the fisherfolks are back on the shore, the platform helps them list their fish produce on the mobile application. The digital platform helps connect buyers and fisherfolks directly. The two stakeholders can negotiate price, quantity based on the quality and specification of the produce and seal the deal and generate the formal invoice (captured in Fig. 4). The consignment contains the invoice with the QR code. Upon scanning the QR code, the entire traceability and tracking of the fish produce can be accessed. For instance, it offers answers to the questions like when it was caught in the deep seas, who were the fisherfolks, what is the specification of the fish, when did it reach the shore, etc. This includes information on fish quality (perishability factor) and hence increases trust and traceability in the supply chain, especially to the buyer (captured in Fig. 5).

**Fig. 6:**
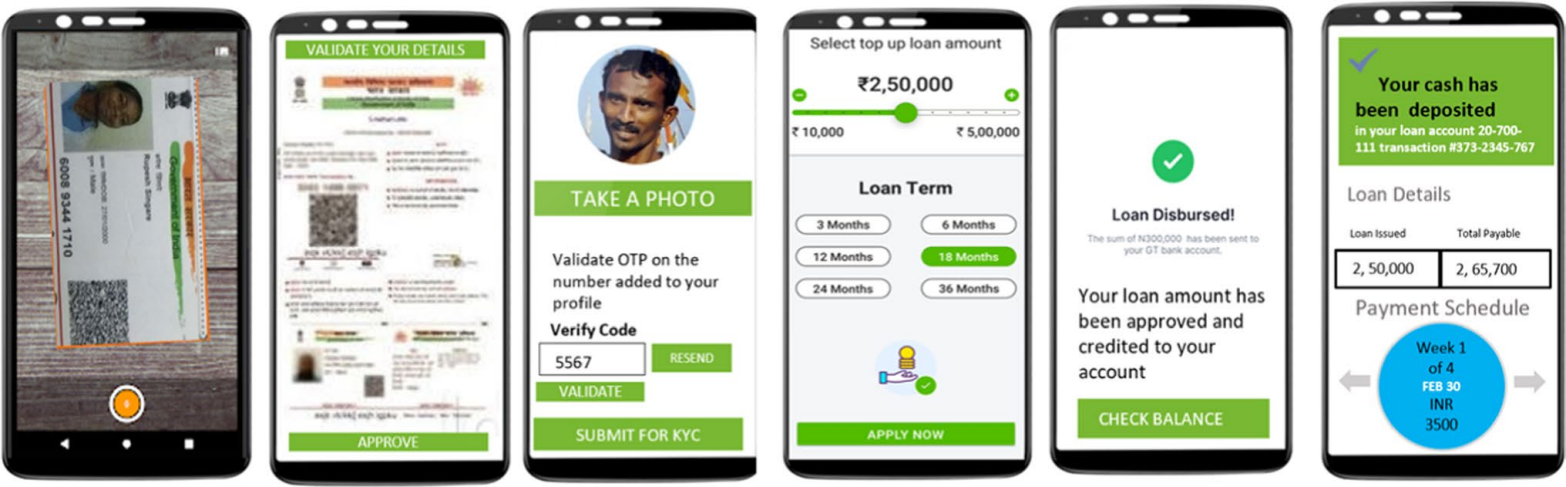
Livelihood Support through Microfinance

In addition, Numer8 also provides livelihood support. The mobile applications collect fisherfolks profile data, their trip details, and catch details, and supplies them to suitable microfinance institutions through their credit rating model (as shown in Fig. 6). This aspect is extremely important from the perspective of larger participation and resilient ecosystem as fishery business is highly volatile. Formal institutions are reluctant to provide loans to unorganized fisherfolks in developing country such as India (Barua et al., 2012). This is mainly because there is lack of credible data and it is extremely difficult for lending institutions to assess the creditworthiness of fisherfolks in large numbers scattered across the coastline.

**Fig. 7:**
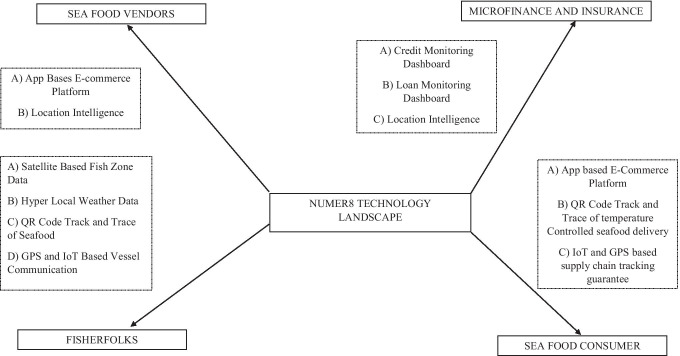
Technology landscape

The fisherfolks, owing to their volatile business, are often constrained by working capital issues and irregular payment cycle. This increases their dependence on local money lenders who charge high interest rates (5% to 6%). This support provides an opportunity for the economically disadvantaged fisherfolks to participate in the business landscape and as a result earn more money in the process coupled with traceability of the entire supply chain. The traceability and quality assurance allows more buyers to participate in the model. This business model supports other vendors such as logistic partners and cold storage container providers in the same platform while pricing and other formalities are carried out between fisherfolks and buyers in the process. To summarize the entire technology landscape (captured in Fig. 7), fisherfolks are provided with satellite-based fish zone data, hyper local weather data, QR code track and trace of seafood, GPS and IoT based vessel communication and app-based livelihood support system.

**Fig. 8:**
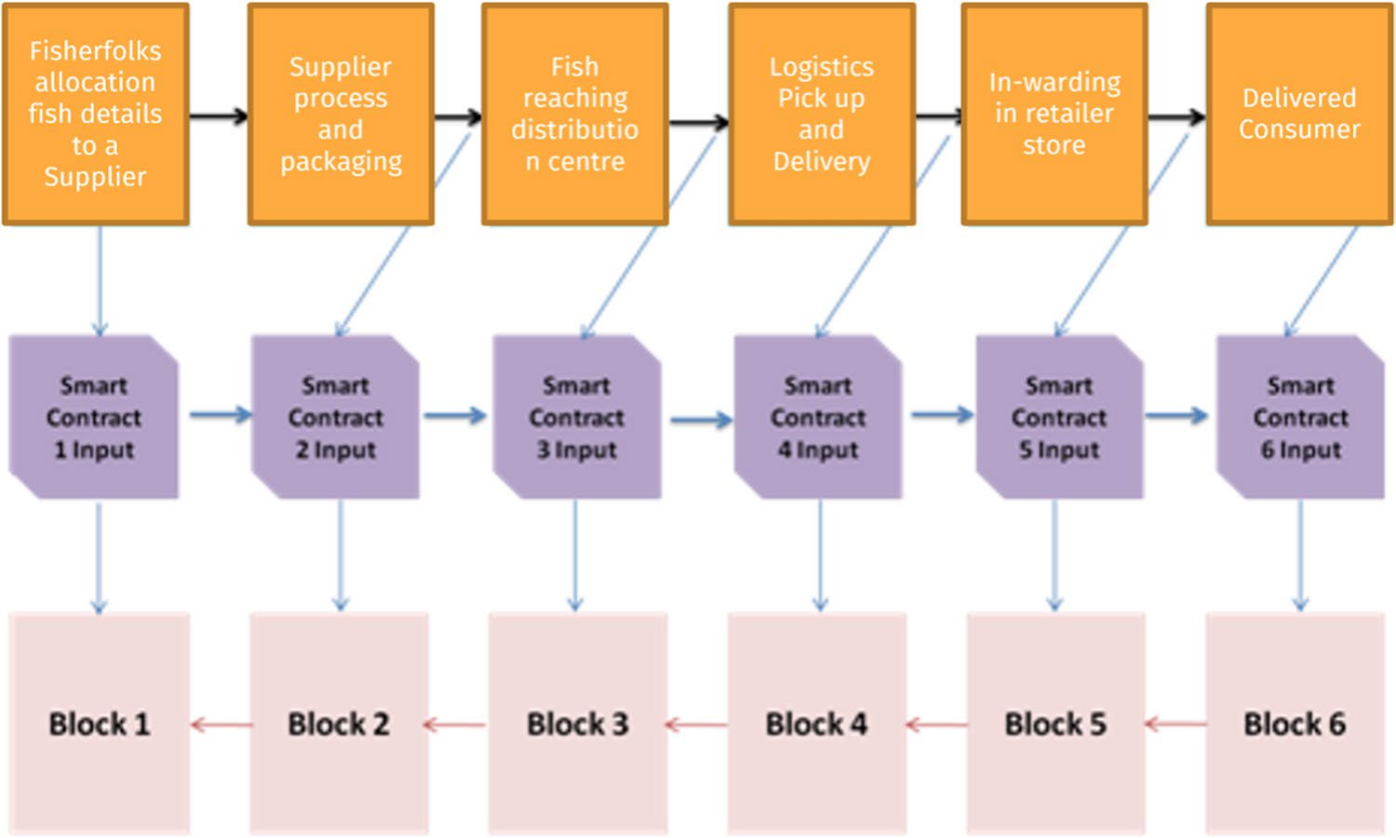
Blockchain technology design

For the seafood consumer, Numer8 introduced the Blockchain technology (Disruptive Technology 2). Blockchain, coupled with data sensors (IoT and satellite), can improve quality, and safety in the fresh seafood supply chain. It can significantly improve industries margin on fresh produce such as fish, by reducing waste almost by 50%. Blockchain system would enable companies to guarantee transparency which will significantly increase consumer trust and brand value. One of the main advantages of blockchain is that once information is added to the blockchain from the data sensors, it is distributed within the network and it becomes immutable. It cannot be hacked, manipulated, or corrupted in any way. The technology behind data sensors and electronic chips such as RFID seals is evolving rapidly. This evolution allowed companies to attach sensors to goods to track and thereby detect potential failures and fraud. The data received from these sensors with a Blockchain can provide standardization, transparency & traceability to supply chain. This will help seafood industries in minimizing risks by ensuring temper-proof & reliable flows which was missing from the seafood industry for a long time. Numer8 has developed a food supply chain system using Blockchain framework and data science. Sensors will track the status of goods as they move through the supply chain and share this information to a blockchain-based framework which ensures that all of the participants in the chain can access the data in real time and can validate which increases trust between parties. The details of the “blocks” and the potential “smart contract” are presented in Fig. 8.

### Disruptive Technology Integration and Supply Chain Resilience

After the outbreak of COVID-19, it is relevant to understand whether the integration of disruptive technologies lead to supply chain resilience for several reasons. First, the global pandemic has taught us with multiple waves that developing nations need to have robust supply chains for their food sector in order to maintain a steady supply of produce across the regions. Second, the unorganized nature of such sectors exposes the economically weaker sections of the society when there is an external shock such as the global pandemic.

The bottlenecks have been discussed in the previous section and hence policy makers and practitioners need to appreciate the fact that the global pandemic amplified the existing voids in the system. If we observe our first disruptive technology, that is satellite imagery, we found that apart from providing weather related data, it contributes significantly to cost reductions and most importantly reduces the time and risk exposure associated with deep sea fish catching process. Hence, not only it impacts supply chain efficiency (cost reduction), but also contributes towards supply chain responsiveness (search time reduction). This is in addition to supply chain risk mitigation at the supplier level. Hence, only observing from the supplier’s (fisherfolks) perspective, they are (fisherfolks) definitely better off in all the dimensions related to its activities before the sale of fish commence.

Fish being a perishable produce and due to lack of trust between buyers and sellers, it is always subjected to quality checks. This disagreement with respect to quality determination leads to volatility in the price offered at different points in the value chain. This severely impacts the income generation potential of the economically disadvantaged fisherfolks and also the threat of being cheated at the consumer level due to non-conformance of quality. Hence, tracking and tracing of fish produce are necessary. Therefore, not only the IoT device in fishing boats (tracking) is important but also the QR code (tracing) ensures that proper quality monitoring is implemented and the information can be accessed. This is only possible through IoT integration with Satellite and QR Code (digital platform).

The moment the consumer is able to verify the perishability dimension of the produce, prices will tend to move towards the fair price model since consumers are able to determine the right quality and hence pricing will move towards an equilibrium point agreeable to both the buyer and the seller. This price will ensure producer surplus at the level of fisherfolks with quality assurance at the end consumer level. This reduces both demand side and supply side bottlenecks to a great extent, thereby leading to supply chain resilience as it is more immune to shocks. Any shock, if it impacts the upstream supply, will get reflected in the traceability dimension and hence prices will stabilize at the right level.

However, when it comes to payment information and its transparency at the entire supply chain level, blockchain is the only solution. Therefore, with smart contracts in place, all players in the supply chain will be able to observe the prices, quantities and the entire movement of produce to the final consumer. This will enhance trust and more players would be willing to adopt the digital platform and the entire supply chain would move towards being organized and more immune to both internal and external shocks. We believe that this would improve supply chain resilience and should hold true for any emergency situations such as global pandemic and other natural calamities.

## Discussion

**Fig. 9:**
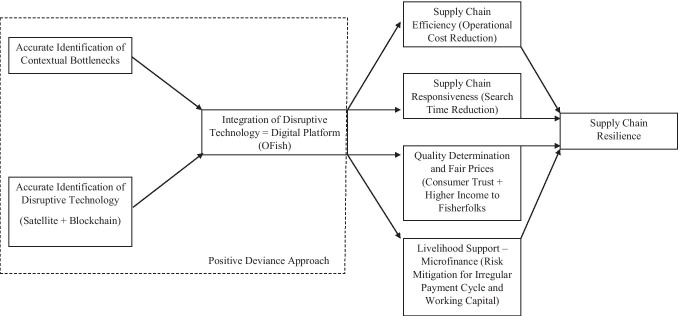
Proposed Framework for Supply Chain Resilience through Disruptive Technology Integration

The important aspect here is to understand how Numer8 adapted, integrated and implemented disruptive technologies to achieve resilience. We present our framework in Fig. 9. Many practitioners often endorse a need-based approach where key decision makers list out different needs and problems and then impose a solution to address those needs (Albanna & Heeks, 2019). However, such an approach works best when the need for learning and adaptation to the local environment is absent. In our case, Numer8’s success largely depended on a bottom-up approach that required firms’ such as Numer8 to capitalize on the strengths of the local communities, often termed as the “asset-based” approach. These “assets” can be contextual knowledge, inherent capabilities due to presence of working in similar environments for years, and resources that help in building those capabilities. Positive Deviance is a similar “asset-based” approach (Albanna & Heeks, 2019). Most of these approaches are uncommon in order to achieve superior results. The first important task of Numer8 was to understand the local challenges or bottlenecks in the fishery sector. This is critical as firms may end up investing in non-value-added infrastructure without achieving desired results. One of the key contextual observations in the fishery sector is the lack of transparency across the supply chain because of which end consumers suspect the quality of fish and the fisherfolks are burdened with unfair prices and information asymmetry on the actual demand. Hence, as “positive deviants”, Numer8 had to bring in a digital platform that could track and trace the entire supply chain and, in the process, bring buyers and sellers together.

However, the next activity was to decide on the technology to track and trace. With the help of satellite technology and GPS system on fishing boats and logistic vehicles, coupled with the IoT devices, Numer8 was able to track and trace the entire supply chain. Further, from the perspective of addressing the issues of fisherfolks, satellite technology was instrumental in reducing the search cost (efficiency) and search time (responsiveness) as explained in Fig. 9. Also, last mile consumers were sceptical with quality of the fish produce and therefore the ability to track and trace through Satellite with the help of IoT and GPS was key in addressing the identified bottlenecks in the system. This approach was not easy as it involved acceptance of the digital platform from key stakeholders based on the perceived future benefits (Sengupta et al., 2021). The issue of unfair prices and transparency was addressed by integrating Blockchain. This was achieved through QR code, IoT devices and its integration with blockchain applications combined together in the digital platform. This increased overall transparency and trust. Numer8 used the “asset-based” positive deviance approach by capitalizing the resources of logistic partners, seafood vendors, and micro-finance institutions, which provided support to fisherfolks and the entire value chain at large. The overall benefits highlighted in the results would have been difficult to achieve unless bottleneck identification and technology integration was not carried out effectively.

## Conclusion

Our paper explains how disruptive technology can be used to address contextual challenges of food supply chain in developing markets. Through the positive deviance approach and taking India as the empirical context, our case study with Numer8 attempts to add value to fisherfolks by integrating satellite imagery and blockchain technology. The implementation comes at the backdrop of the global pandemic which has severely affected the global supply chain and in particular food supply chains in developing nations.

We found that disruptive technology such as satellite imagery and IoT helps fishing supply chain to reduce their search cost and increase consumer (buyers) trust on prices of different varieties of fish given specific information on the perishability factor. Most importantly, this disruptive technology helps improve fishermen income by transferring the benefits from middlemen to suppliers. Finally, it highlights the relevance of such technology in smooth functioning of supply chain in the event of future pandemics, thus contributing to supply chain resilience by bringing together buyers and suppliers under one digital platform.

The blockchain technology framework increases trust and transparency and with the help of Satellite and IoT, it adds the traceability and quality monitoring dimension in the system thus improving the supply chain resilience in the value system. We propose a framework as a means to achieve supply chain resilience in the emerging market context for the fishery sector with the help of disruptive technologies. We specifically wish to highlight the importance of positive deviance as an effective approach to tackle contextual bottlenecks in developing regions specifically when the supply chains are unorganized (fishery, agriculture, handicraft and handlooms), serves as a backbone to the economy (food sector), and most importantly in situations where technological interventions are difficult owing to the reluctance to adopt such technologies at a large scale where stakeholders are economically disadvantaged.

### Research Implications

Our paper has important research implications. First, our paper extends the emerging research on the application of technology in fish supply chains (Karlsen et al., 2011). Past literature highlights the technological side of the application. However, our paper points out the contextual challenges of stakeholders in such developing economies and the relevance of the right kind of technology to benefit all stakeholders in the process. Second, the paper makes a modest attempt to use positive deviance as a framework to understand how firms, instead of people and communities, could redesign its supply chain by using resources that are already available in the market to address contextual challenges in developing nations in comparison to its peers and competitors. By doing so, it adds new information on the very scant work on fish supply chain in information systems and operations management literature (Abedi & Zhu, 2017). The positive deviance approach essentially captured how the economic benefit for the disadvantaged stakeholder because of technological intervention could be improved by actions taken by firms and its associated stakeholders. Third, our paper strongly contributes to the current global pandemic (COVID-19) studies by extending the discussion on information processing, supply chain resilience and network characteristics of supply chains for risk mitigation (Gu et al., 2020; Ivanov & Dolgui, 2020; Li et al., 2020; Wong et al., 2020). Our study extends the discussion on supply chain resilience during COVID-19 by bringing in issues of information technology integration, stakeholder management in a fairly unorganized setup and most importantly how the design of network which includes how different stakeholders in the value chain are linked to the digital platform thereby ensuring better traceability and transparency. Lastly, this paper highlights the role of technology as an enabler to promote socially responsible businesses and contributes to the discussion on “poor as suppliers” (Shivarajan & Srinivasan, 2013; Sodhi & Tang, 2014). Our findings emphasize how larger participation of “poor as suppliers” can be achieved if the right mix of technology in integrated in the supply chain so as to benefit them economically. We found that the digital platform of Numer8 backed by satellite imagery and blockchain technology was not only instrumental in improving the overall resilience of the supply chain but also ensured that due to traceability of the entire value chain, both consumers and poor fishermen stand to benefit for fair price for the actual quality of the fish produce.

### Practice Implications

This study has important practice implications. First, we found that traceability of fish produce through the blockchain concept not only improved the quality determination factor of the produce in the supply chain but also increased the trust between stakeholders. This has immense application to other perishable produce such as the agricultural sector in developing nations. One must note that in developing countries such as India, China, Myanmar etc., the dependence on agricultural and fish produce is significant (livelihood of stakeholders and as a source of food to the entire population) while both being perishable. As a result, scaling up such innovation could reduce food wastage, increase earning opportunities of upstream players and most importantly the consumer surplus could improve in terms of determining the right price for a certain product. Second, the digital application and satellite imagery together presents itself an important asset for fisherfolks, transportation agency and wholesale buyers in many ways. While the search cost is reduced due to satellite imagery, the digital application reduces the total duration of the fish produce being idle at the shore since buyers and transporters are automatically informed regarding the quantity, quality and price of the produce. Similar applications in agricultural sector are needed as poor farmers are often unable to sell their produce to prospective buyers and they incur significant expense for transporting their produce to the local market. Hence, it has tremendous importance to better income earning opportunities for economically disadvantaged stakeholders in the upstream food supply chain. Third, the presence of digital platform allows micro-entrepreneurs to get involved in the business model by providing products such as cold storage containers, fishing nets, cold storage centres among others. It also allows offering services as a logistics partner thereby strengthening the stability of the network which in turn would attract all stakeholders (sellers, buyers and other vendors) to adopt and accept this business model. The higher the participation, the level of trust through the entire value chain would improve. This has far reached implications, from the perspective of development, employment and earning opportunities, to the local economy in particular and also reducing the voids in the current system.

### Social Implications

The social implications from this business landscape are noteworthy. First, economically disadvantaged fisherfolks will earn more due to 1) reduction of search cost and time achieved due to satellite, GPS and relevant IoT devices attached to the fishing boats 2) reduction of middlemen connecting buyers and sellers leading to accurate information regarding demand and 3) incomplete information from downstream players leading to unfair prices being offered to fisherfolks. The bargaining power of the downstream player (middlemen) was higher as fish was a perishable produce and fisherfolks had to sell the produce at a lower rate with insufficient information on prices and demand originating from last mile consumers. Second, the presence of digital platform has ensured that fisherfolks can take the help of critical resources such as microfinance, cold storage containers, logistic partners etc. The presence of microfinance helps fisherfolks manage their irregular working capital which is normally caused due to delay in payment cycle. The availability of cold storage containers helps them to sell at the right price instead of selling at lower prices as the produce is perishable. The combination of cold storage containers and logistic partners (and the ability to track and trace) ensures that buyers will pay the right price as quality is determined in the entire value chain. This is leads to better sales value, ability to cater to the demand without compromising on quality and most importantly generates livelihood by providing employment or business opportunities through this process. Third, the long-term implications, for such resilience driven innovation in food supply chain, are reduced inequalities (SDG 10), responsible consumption and production (SDG 12) and zero hunger (SDG 2) especially in developing market context.

### Limitations and Future Scope

Our paper has limitations and opens up areas for future research. First, our case study is based on one organization who has implemented disruptive technology in the fish supply chain context in India. Owing to the already stated complexity of being unorganized and dealing with stakeholders from different economic conditions, it is very difficult to find such organizations across India implementing the same in the fishery sector. Hence, future studies could document the journey of other organizations implementing similar disruptive technology in the fishery sector. Second, although the case organization has implemented similar projects in other developing countries such as Sri Lanka, etc., due to the global pandemic and the subsequent travel restrictions, we could not validate our findings in similar contexts. Although, the core learning’s would remain somewhat same, due to cultural differences and contextual conditions, the degree of complexities may differ in different regions depending on the existing infrastructure, the ease of implementing and adopting such disruptive technology and the degree to which different stakeholders needs to be taken into confidence for being part of this initiative. Third, implementing a true blockchain technology is always difficult as it involves satisfying different criterion such as distributed ledger, immutable, unanimous, programmable, secure and anonymous. The objective of our paper was not to verify all the properties of the blockchain technology and how at the back end it is implemented. Future studies could look into whether the entire criterion is met for organizations who claim that they are using blockchain technology.
